# Vulvar Varicosities and Pelvic Venous Disorders in Nongravid Women: A Case Series

**DOI:** 10.3390/jcm15041558

**Published:** 2026-02-16

**Authors:** Benjamin Daniel, Jennifer Dennison, John Regan

**Affiliations:** Department of Radiology, Wake Forest School of Medicine, Winston Salem, NC 27101, USA

**Keywords:** pelvic venous disorders, vulvar varicosities, stenting, embolization, pelvic exam, pelvic pain

## Abstract

**Background/Objectives**: The authors hypothesize that some vulvar varicosities are due to and can be treated by addressing underlying pelvic venous disorders (PeVDs). The purpose of this single center retrospective study is to evaluate vulvar varicosity resolution following treatment of an underlying PeVD. **Methods**: This study is a single center, retrospective case series from 2010 to 2025 of all patients evaluated in a single vein clinic with vulvar varicosities confirmed by examination and/or imaging, most commonly CT abdomen and pelvis with contrast. Inclusion criteria were presence of vulvar varicosities, evidence of an underlying PeVD, treatment with either left ovarian vein embolization or left iliac stenting, and at least one month of follow-up. PeVD was defined as a combination of suggestive imaging findings (left ovarian vein dilation or left common iliac compression) combined with associated symptoms including pelvic pain and pelvic fullness. Exclusion criteria included prior intervention for PeVDs, other vascular pathologies such as vascular malformations, incomplete documentation, and inaccessible imaging. **Results**: A total of 18 women with an average of 44 years of age met inclusion and exclusion criteria for the study. Thirteen patients (72.2%) presented with lower extremity varicosities at the same visit. Fifteen patients were multiparous at the time of presentation with a para status averaging 2.5. Ten patients (55.6%) had left ovarian reflux confirmed venographically and received ovarian vein embolization. Preoperative or intraoperative left ovarian venous diameter averaged 7.8 mm. Seven patients (38.9%) had left common iliac vein compression and received self-expandable left common iliac venous stenting. Preoperative CT suggested compression and all patients had intraoperative intravascular ultrasound (IVUS) prior to stenting with an average stenosis of 75.9%. One patient had both pathologies and received both treatments. No patients underwent right ovarian vein embolization nor had venographic evidence of right ovarian reflux. A total of 16 out of 18 patients (88.9%) had complete resolution of PeVDs. One patient had partial response for pelvic pain at one month of follow-up. Another patient had recurrence of pelvic pain symptoms and is being worked up for Nutcracker syndrome. All patients had resolution of their vulvar varicosities on follow-up examination. **Conclusions:** Vulvar varicosities may be indicative of an underlying PeVD. Vulvar varicosity resolution is associated with PeVD treatment in this case series. Therefore, vulvar varicosities are an important physical exam finding in pelvic examination and referral to a vein specialist should be considered. Additional higher powered, prospective, and randomized studies are indicated to further evaluate this relationship.

## 1. Introduction

Vulvar varicosities can occur in up 4% of women [1]. It has a higher incidence in women with additional lower extremity varicosities reaching 34% and pregnant women ranging from 10 to 22% depending on the source [2,3]. The mechanism of vulvar varicosity formation during pregnancy has been hypothesized to be from compression of the gravid uterus on pelvic venous outflow and progesterone mediated venous dilation [3]. Multiparity worsens symptoms due to multiple periods of stress upon the local perineal valves. Vulvar varicosities that persist beyond pregnancy have a similar mechanism to peripheral varicosities in that the valves of draining veins from the perineum have become incomplete causing blood to pool in the pelvic floor and labia [4].

Pelvic venous disorders (PeVDs) account for as much as 30% of chronic pelvic pain and up to 8% of women may experience some form of this condition [5]. The American Vein and Lymphatic Society International Working Group on PeVDs has helped to standardize discussion and terminology related to this condition with the Symptoms-Varices-Pathophysiology (S-V-P) classification system [6]. It is important to evaluate vulvar varicosities using this overarching schema. Patients with symptoms related to vulvar varicosities fall into the S2 category of chronic pelvic pain of venous origin. They may or may not also present with the S3 category of extra-pelvic symptoms of venous origin. Similarly, their variceal location puts them in the V2 category of pelvic varicosities. If there are pelvic escape veins and associated lower extremity varicosities, then they would be considered in the V3 category of pelvic-origin extra-pelvic varicosities. Pelvic escape veins are collaterals connecting the pelvis to the superficial veins of the lower extremity. Finally, the underlying pathophysiology can be due to multiple anatomic causes most commonly relating to obstruction of venous return from left iliac compression, occlusion or thrombosis (May-Thurner spectrum) as well as venous reflux, most commonly from an incompetent left ovarian vein.

While the categorization of vulvar varicosities within the greater context of PeVD and S-V-P is important for clinical contextualization, it is easy to get lost in the details of the classification system. Therefore, providers interfacing with chronic pelvic pain patients who may have underlying venous disorders must keep a foundational understanding of how varicosities generally develop. A more intuitive model can be imagining the patient in the upright position with their venous system simplified as a column of blood. The effects of gravity are constantly acting on this column and competent valves are the mechanistic safeguard preventing blood from flowing with gravity in the wrong direction, away from the heart [7]. When this process breaks down, blood can pull in multiple reservoirs, most commonly the pelvis and lower extremities. The S-V-P system is designed to evaluate patients with pelvic pooling as described while the CEAP (Clinical-Etiology-Anatomy-Pathophysiology) classification is a separate system used for lower extremity veins and is beyond the scope of this paper [8].

Vulvar varicosities during pregnancy are often temporary since venous compression from the uterus and progesterone secretion are both self-resolving with delivery. However, multiple pregnancies can injure valves and are a risk factor for persistent vulvar varicosities. In contrast, vulvar varicosities outside of pregnancy can be thought of as the tip of the iceberg, where there may be PeVD hiding beneath the surface such as ovarian venous reflux or iliac compression. Unfortunately, the literature on the treatment of vulvar varicosities is limited without substantial prospective data. “Top-down” approaches and “bottom-up” approaches have been hypothesized, where interventionalists start by treating the most cranial obstruction or start by directly injecting the associated varices [9].

Here, it is hypothesized that focusing on a top-down approach for nongravid patients with vulvar varicosities will be an effective treatment strategy for vulvar varicosities. If the incompetent or obstructed vein that is increasing the venous pressure within the pelvis is eliminated or fixed, then it follows pathophysiologically that the varicosities themselves should no longer be pressurized.

## 2. Materials and Methods

This study is a single center, retrospective case series from 2010 to 2025 of all patients evaluated in a single vein clinic with vulvar varicosities confirmed by examination and/or imaging. This research/study was approved by the Institutional Review Board at Atrium Health Wake Forest Baptist retrospective umbrella protocol, number IRB00050858, dated March 2023. All patients signed an informed consent with stipulations for use in retrospective research.

Inclusion criteria were presence of vulvar varicosities, evidence of an underlying PeVD, treatment with either left ovarian vein embolization or left iliac stenting, and at least one month of follow-up. PeVD was defined as a combination of suggestive imaging findings (left ovarian vein dilation or left common iliac compression) combined with associated symptoms including pelvic pain and pelvic fullness. Left ovarian venous dilatation was defined as greater than 6 mm [10]. This was assessed either with preoperative CT or intraoperative venography. Venography also confirmed ovarian venous stasis or regurgitation, which was a necessary finding prior to intervention. Left iliac compression was subjectively assessed on pre-operative imaging, but was quantified on all patients with intraoperative IVUS. Iliac compression on IVUS was defined as greater than 50% stenosis [11]. Additional venographic findings such as retrograde flow into the left internal iliac vein or left ascending lumbar vein were useful additional findings suggesting stenosis, but IVUS was the definitive variable used to confirm the need for venous stenting.

Exclusion criteria included prior intervention for PeVD or other venous intervention that could alter flow into and out of the pelvis, other vascular pathologies that would alter venous flow such as vascular malformations, incomplete documentation, and inaccessible imaging. All premenarchal patients were also excluded.

Artificial intelligence software was not used at any point. Since this is a small case series and is observational in nature, no statistical software was used. This study relies on descriptive statistics. The primary outcome, resolution of vulvar varicosities, was defined as complete resolution of varicosities on follow up physical examination. Complete resolution of PeVD was defined as complete resolution of visible varicosities combined with complete resolution of the clinical symptoms common to PeVD including pelvic fullness and pelvic pain.

## 3. Results

In the defined study period, 543 patients received venographic interventions for PeVD. However, very few patients had complete pelvic examinations or a description of an assessment of vulvar varicosities. Additionally, multiple patients with appropriate documentation did not have available imaging due to an institutional EMR shift. Ultimately, a total of 18 women with an average of 44 years of age met inclusion and exclusion criteria for the study (Table 1). Fifteen patients were multiparous at the time of presentation with a para status averaging 2.5.

Ten patients (55.6%) had left ovarian reflux confirmed venographically and received ovarian vein embolization. Embolization was performed with pushable Nestor (Cook Medical, Bloomington, Indiana) coils. The angiographic endpoint was occlusion of the vein with resolution of reflux. Patients with ovarian venous reflux had a preoperative or intraoperative left ovarian venous diameter averaged 7.8 mm.

Seven patients (38.9%) had left common iliac vein compression and received Abre (Medtronic, Independence, Ohio) self-expandable left common iliac venous stenting. Preoperative CT suggested compression on all of these patients. However, due to inherent limitations in CT for iliac stenosis quantification, all patients had intraoperative intravascular ultrasound (IVUS) prior to stenting with an average stenosis of 75.9% [12]. All patients had less than 20% residual stenosis following stenting.

One patient had both pathologies and received both treatments. No patients underwent right ovarian embolization nor had venographic evidence of right ovarian reflux. A total of 16 out of 18 patients (88.9%) had complete resolution of PeVD. One patient had partial response for pelvic pain at one month of follow-up reported as a subjective decrease in symptoms of approximately 50%. Another patient had recurrence of pelvic pain symptoms and is being worked up for Nutcracker syndrome. All patients had resolution of their vulvar varicosities (Table 2).

Thirteen patients (72.2%) presented with lower extremity varicosities at the same visit. Of these patients, 2 patients had resolution of lower extremity varicosities following PeVD. Both patients had documented pelvic escape varicosities supplying their lower extremity varicosities. The remaining patients had their lower extremity varicosities treated with sclerotherapy and ablation separately.

There were no complications related to these procedures. All patients had the procedures done as outpatients.

## 4. Discussion

This is a small retrospective case series without statistical significance meant for future hypothesis generation. Eighteen patients with nongravid, persistent vulvar varicosities demonstrates 100% resolution of varicosities using a top-down approach to treat the patient’s underlying PeVD. The underlying lesions causing the PeVD were related to left common iliac compression/occlusion and/or left ovarian vein incompetence. The majority of patients had prior pregnancies and as well as concomitant lower extremity varicosities. The minority of patients with lower extremity varicosities had resolution following PeVD treatment alone, and the two who did had documented pelvic escape veins supplying their lower extremity varicosities.

This study ultimately supports the initial hypothesis that treating the causative venous pathology leading to increased pelvic venous pressure will improve vulvar varicosities and is in alignment with the pathophysiological principles presented by the S-V-P classification system put forth by the American Vein and Lymphatic Society International Working Group on PeVDs. It is not surprising that treating venous hypertension in one reservoir does not fix an underlying pathology in another reservoir. Namely, treating pelvic varicosities does not necessarily fix lower extremity varicosities which are instead usually driven by great saphenous and small saphenous insufficiency.

The single patient who had recurrence of pelvic pain combined with flank pain also had microscopic hematuria and left renal stenosis evidenced venographically with left renal hilar collateral filling. Therefore, the recurrence of symptoms could be from not addressing the true “top” of the patient’s venous pathology. In this case, embolizing the ovarian vein does not effectively resolve venous hypertension from renal compression. A second patient had partial response for pelvic pain at one month of follow-up and is expected to continue to improve over time.

None of the patients who met criteria for inclusion in this study had vulvar varicosities without evidence of an underlying PeVD, which goes against some of the existing literature [13]. This could be due to the small sample size of these patients. It also raises the question of true persistence of vulvar varicosities outside of pregnancy in the absence of an underlying venous pathology increasing pressure in the pelvic reservoir, whether it is driven by ovarian insufficiency or iliac compression. Regardless, should a top-down approach fail or an underlying venous pathology not be identified, then direct sclerotherapy of vulvar varicosities should still be considered. This study is roughly aligned with the previous literature demonstrating greater than 83% resolution with a top-down approach used for iliac stenting in patients for vulvar varicosities, though the literature is limited [9].

This case series has several important limitations. It is a small sample size. It is not prospective or randomized. There is a lack of comparative data including no control group. Statistical significance is not presented. There are several candidates for confounding variables including concomitant lower extremity venous intervention. Measuring vein diameter during pressurization from venography can overestimate diameter due to pressurization of a capacitance vessel. A clinical assessment tool to further quantify the patient’s symptoms was not used producing a lack of a standardized, objective outcome measurement. Physical examination pictures before and after treatment were not saved. Patients were evaluated with multiple pathologies causing vulvar varicosities.

The volume of patients is less than expected based on the volume of patients from 2010 to 2025 and the reported incidence of 4%. This, combined with the lack of pelvic examination documentation on a large number of patients, raises the possibility that patients with vulvar varicosities may have gone underreported, and also introduces a potential selection bias to the patients presented, meaning that the patients may not be truly representative of this pathophysiology.

## 5. Conclusions

Vulvar varicosities, particularly in nongravid women, are an important physical exam finding when evaluating patients with pelvic pain and pelvic floor dysfunction. While preliminary and meant for hypothesis generation, this small case series demonstrates varicosity resolution is associated with utilizing a top-down approach treating the patient’s underlying PeVD that may be responsible for their varicosities. This is a limited case series with numerous limitations and possible confounding variables, but it highlights the importance of involving a vein specialist in these situations and the need for further prospective, randomized data to further assess treatment options for these patients.

## Figures and Tables

**Table 1:** Patient characteristics.

Patient Characteristics	Percentage
Patients with Iliac Compression (%)	38.9
Patients with Ovarian Reflux (%)	55.6
Patients with Both Ovarian Reflux and Iliac Compression (%)	5.5
	Range, Mean
Patient Age (Years)	21–68, 44
Iliac Vein Compression, Intraoperative IVUS (%)	61–100, 75.9
Left ovarian vein diameter (mm)	4–10, 7.8
Gravida Status	0–8, 3.3
Para Status	0–6, 2.5
Follow-Up Time (Months)	2–73, 12.25

**Table 2:** Patient outcomes.

Patient Outcomes	Percentage, Total Number
Vulvar Varicosity Resolution	100, 18
Pelvic Pain Resolution	88.9, 16
Lower Extremity Varicosity Resolution	11.1, 2
Technical Success	100, 18
Complication Rate	0, 0

## Data Availability

The original contributions presented in this study are included in the article. Further inquiries can be directed to the corresponding author.
